# National Blood Pressure Education Month — May 2013

**Published:** 2013-05-10

May is National Blood Pressure Education Month, dedicated to increasing awareness and educating patients and the public about hypertension and its impact on health. Hypertension, also known as high blood pressure, is a leading risk factor for cardiovascular disease and a major cause of morbidity and mortality (1). In the United States, nearly one in three adults (67 million persons) has hypertension. More than half of persons with hypertension do not have it under control, and 14 million adults with uncontrolled hypertension do not know they have hypertension (2). Hypertension contributes to nearly 1,000 deaths per day and costs the nation $47.5 billion in direct medical expenses each year (1).

Patients can achieve greater hypertension control by taking their medications as directed, measuring their own blood pressure, and eating a lower-sodium diet. Health-care providers and systems can use electronic health records, blood pressure monitoring, and a team-based care approach to help improve their patients’ hypertension control (3).

Million Hearts, a U.S. Department of Health and Human Services initiative led by CDC and the Centers for Medicare and Medicaid Services, is focusing efforts to prevent 1 million heart attacks and strokes by 2017. Million Hearts is working to reduce hypertension by 1) educating health-care professionals, health systems, insurers, employers, and individuals about the link between blood pressure control and health, and 2) empowering all persons to make healthy choices, such as preventing or quitting tobacco use and reducing salt (sodium) and trans fat consumption, to decrease the number of persons who need medical treatment and to prevent heart attacks and strokes. Additional information about Million Hearts is available at http://millionhearts.hhs.gov. Additional information about hypertension is available from CDC at http://www.cdc.gov/bloodpressure.
